# Nitric Oxide Involvement in Cardiovascular Dysfunctions of Parkinson Disease

**DOI:** 10.3389/fphar.2022.898797

**Published:** 2022-07-11

**Authors:** Marli Cardoso Martins-Pinge, Lorena de Jager, Blenda Hyedra de Campos, Lorena Oliveira Bezerra, Pamela Giovana Turini, Phileno Pinge-Filho

**Affiliations:** ^1^ Departamento de Ciências Fisiológicas, Universidade Estadual de Londrina—UEL, Londrina, Brazil; ^2^ Departamento de Ciências Patológicas, Universidade Estadual de Londrina- UEL, Londrina, Brazil

**Keywords:** arterial pressure, heart rate, hypotension, 6-OHDA, NO-synthase, parkinsonism

## Abstract

Parkinson’s disease (PD) is characterized by the loss of dopaminergic neurons in the substantia nigra, causing motor changes. In addition to motor symptoms, non-motor dysfunctions such as psychological, sensory and autonomic disorders are recorded. Manifestations related to the autonomic nervous system include the cardiovascular system, as postural hypotension, postprandial hypotension, and low blood pressure. One of the mediators involved is the nitric oxide (NO). In addition to the known roles such as vasodilator, neuromodulator, NO acts as an important mediator of the immune response, increasing the inflammatory response provoked by PD in central nervous system. The use of non-specific NOS inhibitors attenuated the neurodegenerative response in animal models of PD. However, the mechanisms by which NO contributes to neurodegeneration are still not well understood. The literature suggest that the contribution of NO occurs through its interaction with superoxides, products of oxidative stress, and blocking of the mitochondrial respiratory chain, resulting in neuronal death. Most studies involving Parkinsonism models have evaluated brain NO concentrations, with little data available on its peripheral action. Considering that studies that evaluated the involvement of NO in the neurodegeneration in PD, through NOS inhibitors administration, showed neuroprotection in rats, it has prompted new studies to assess the participation of NOS isoforms in cardiovascular changes induced by parkinsonism, and thus to envision new targets for the treatment of cardiovascular disorders in PD. The aim of this study was to conduct a literature review to assess available information on the involvement of nitric oxide (NO) in cardiovascular aspects of PD.

## Introduction

Parkinson’s disease (PD) is the second most common neurodegenerative disease and affects approximately 1% of the world population aged over 65 years (Lau and Breteler, 2006). It is characterized by the degeneration of dopaminergic neurons in the substantia nigra pars compacta (SNpc) and formation of α-synuclein aggregates, called Lewy bodies. When the neurodegenerative process reduces dopamine levels by about 60%, motor symptoms such as bradykinesia, rigidity and tremor at rest are manifested, mainly (Dauer and Przedborski, 2003). PD also has non-motor symptoms present in the most basal stages of the disease, such as sensory, neuropsychiatric and autonomic disorders, known as dysautonomias (Sian et al., 1999; Rodriguez-Oroz et al., 2009; Schapira et al., 2017)

The worldwide occurrence of Parkinson’s Disease (PD) is estimated between 5 and 35 new cases per 100 thousand inhabitants. The age of onset is usually around 50 years of age, with a higher incidence after the sixth decade of life (Simon et al., 2020). There is a higher prevalence in males, with 134 cases in men and 41 in women per 100,000 inhabitants, aged between 50 and 59 years (Pringsheim et al., 2014). There is also evidence that PD is more prevalent in postmenopausal women compared to premenopausal women of similar age (Ragonese et al., 2006).

With its idiopathic etiology, it is believed that genetic issues are linked to the existence of genes that favor the development of the disease, but acting indirectly. Environmental factors are linked to PD patients who live in rural areas, use well water and are more exposed to pesticides and herbicides (Teive, 2005). Regarding the contribution of brain aging, it would be related to the prevalence of age, associated with progressive neuronal loss (Pereira and Garrett, 2010).

The cardinal signs of PD are: bradykinesia and oligokinesis, observed by slowness and reduction of movements in quantity and amplitude; generalized stiffness; resting tremor and postural instability (Sung and Nicholas, 2013). As the disease progresses, other motor symptoms can be identified, such as reduced facial expression; micrography; disorders in oral communication and swallowing, difficulty in gait, which include freezing, difficulty in changing direction and in overcoming obstacles; postural changes, with a tendency to bend the limbs and respiratory changes, especially due to the decrease in chest expansion (Peterson and Horak, 2016). Motor characteristics of the extrapyramidal region of PD emerge when 30%–50% of the dopamine-producing neurons in the substantia nigra are lost (Fazio et al., 2018). Motor impairment becomes bilateral three to 5 years after the initial diagnosis (Scorza et al., 2001).

Some non-motor symptoms may precede motor dysfunction by several years, such as: neuropsychiatric disorders, sleep disorders, olfactory deficit, anxiety and depression and dysautonomias (Schapira and Tolosa, 2010), but in general, these symptoms occur at any stage of PD, and may vary according to each patient (Jain and Goldstein, 2012).

The autonomic nervous system (ANS), responsible for controlling physiological adjustment parameters, seems to be altered in PD inducing dysautonomias (Azevedo and Cardoso, 2009; Sharabi et al., 2021). A subset of these symptoms are associated with altered function of the ANS, where orthostatic hypotension, hyperhidrosis and gastrointestinal dysfunction are common manifestations in PD that can affect the quality of life of patients and can have an impact even more than the motor symptoms (Teive, 2006). Among the cardiovascular alterations are the orthostatic arterial hypotension (OH), postprandial arterial hypotension, alteration of arterial pressure (BP) variability and, possibly, fatigue and intolerance to physical exercise (Nicaretta et al., 2011; Jain and Goldstein, 2012; Schapira et al., 2017). Those symptoms are quite relevant and impact on the patient’s clinical condition (Sian et al., 1999; Rodriguez-Oroz et al., 2009; Schapira et al., 2017; Balestrino and Schapira, 2020). However, little is know about the origins of those alterations.

Nitric oxide (NO) is a small and highly diffuse molecule that, despite having a half-life of only a few seconds, has an ambiguous action as an important physiological mediator, acting as a vasodilator and immune response mediator, and cytotoxic mediator, exacerbating the inflammatory response caused by PD (Doherty, 2011; Rochette et al., 2013). Considering the involvement of this mediator in the central alterations evaluated in animal models of parkinsonism, the question of its possible involvement in the cardiovascular dysfunctions of PD arises, since NO is a modulator of several neurotransmitter systems, such as dopaminergic, cholinergic and adrenergic (Paredes-Rodriguez et al., 2020). Studies in this direction have been initiated and may provide greater support for the use of new therapies.

## Animal Models of Parkinsonism

The study of Parkinson’s disease (PD) is possible through the induction of the disease in animal models, such as rats and mice. To simulate the main characteristics, pharmacological models such as reserpine and haloperidol, and neurotoxic models such as 6-hydroxydopamine (6-OHDA), 1-methyl-4-phenyl-1,2,3,6-tetrahydropyridine (MPTP), rotenone, paraquat, lipopolysaccharide (LPS) and methamphetamine are utilized (Blum et al., 2001). Neurotoxic models damage the nigrostriatal pathway by infusing neurotoxins into different areas unilaterally or bilaterally in the substantia nigra pars compacta (SNpc), medial forebrain bundle or striatum. 6-OHDA and MPTP are the most used experimental models, since their induction causes the degeneration of 60%–70% of the nigrostriatal pathway, which corresponds to the onset of motor symptoms. Due to the complexity of PD, there is no experimental model that presents all the characteristics of the disease (Duty and Jenner, 2011; Jackson-Lewis et al., 2012).

6-OHDA is unable to cross the blood-brain barrier, so it is administered directly into the SNpc or striatum *via* stereotaxic surgery. The neurotoxin binds to dopamine transporters and is carried to dopaminergic neurons, accumulating in the cytosol and causing the formation of reactive oxygen species that lead to neuronal death. Additionally, 6-OHDA can accumulate in the mitochondria, where it binds to complex I of the electron transport chain and inhibits flow (Blandini et al., 2008).

## Cardiovascular and Autonomic Dysfunction and Parkinson’s Disease

Cardiovascular alterations such as orthostatic hypotension, heart rate variability, modifications in cardiogram parameters and baroreflex dysfunction can appear in both the early and late stages of PD, worsening as the disease progresses. Cardiovascular abnormalities can also appear as a side effect of PD treatment by L-DOPA leading to a decrease in blood pressure aggravating the orthostatic hypotension. This side effect limits the therapeutic use of L-DOPA in geriatric patients with PD and can contribute to the number of hospital admissions (Cuenca–Bermejo et al., 2021). It is estimated that 80% of PD patients have heart rate and blood pressure abnormalities (Gibbons et al., 2017). Orthostatic hypotension affects 40% of PD patients and causes dizziness and syncope, increasing the risk of falls and injuries. These changes seem to be due to noradrenergic cardiac denervation, extracardiac noradrenergic denervation and baroreflex insufficiency (Jain and Goldstein, 2012; Paredes-Rodriguez et al., 2020). Changes in HR appear to be related to dysfunctional parasympathetic responses, while the inability to regulate BP is related to the loss of sympathetic regulation (Jain and Goldstein, 2012).

Heart rate variability assesses the modulation of the autonomic nervous system on the cardiovascular system and works as an indicator of health (Vanderlei et al., 2009). Patients with PD have a reduction in heart rate variability (HRV) and lower LF values ​​than patients in the control group (Solla et al., 2015; Salsone et al., 2016; Strano et al., 2016). Sorensen and colleagues observed attenuated sympathetic activity in patients with PD, with a reduction in the components related to the sympathetic system in HRV (Sorensen et al., 2013). Evidence also shows loss of sympathetic noradrenergic nerves in the heart and kidneys of patients with PD (Goldstein, 2007).

In experimental models, male rats with bilateral induction of the neurotoxin 6-hydroxydopamine (6-OHDA) directly in the SNpc leads to a decrease in baseline parameters of mean arterial pressure (MAP) and heart rate (HR), which are accompanied by a reduction in modulation systolic blood pressure (Ariza et al., 2015b). Baroreflex and chemoreflex responses are altered in these animals (Ariza et al., 2015a). Bilateral injection also caused a drop in the night/day cycle change of heart rate and weakened phenylephrine-induced bradycardia, suggesting a drop in heart rate (Metzger and Emborg, 2019). Furthermore, rats with 6-OHDA parkinsonism show a decreased response to alpha-adrenergic blocker, suggesting an impaired sympathetic vascular synaptic transmission (Ariza et al., 2015b). Bilateral 6-OHDA animals and Prolopa control animals presented a lower cardiovascular compensation during head up tilt, suggesting a possible autonomic impairment in parkinsonism induced by 6-OHDA (Silva et al., 2015). Also, in the 6-OHDA injury model, lower baroreflex sensitivity and decreased number of cardiovascular neurons in the brainstem were observed (Falquetto et al., 2017). Recent literature has described altered central and peripheral mechanisms affecting the feedback-controlled loops that comprise the reflex arc in patients with PD and animal models (Sabino-Carvalho et al., 2021).

In the MPTP model, in mice, attenuation of baroreflex sensitivity was observed, associated with loss of TH + neurons and decrease of catecholamines in the brainstem. There was an increase in HR and a decrease in HRV power. Regarding the frequency domains, an increase in sympathetic tone and a decrease in parasympathetic tone were observed (Liu et al., 2020).

## Nitric Oxide and Parkinson’s Disease

NO is synthesized by nitric oxide synthase (NOS) through the oxidation of L-Arginine. The enzyme is found into constitutive NOS (cNOS), whose activation depends on calcium, and inducible NOS (iNOS), dependent on cytokines. cNOS is expressed at basal levels and has two isoforms: endothelial NOS (eNOS) and neuronal NOS (nNOS). Among the functions of eNOS are vasodilation, inhibition of platelet adhesion and aggregation in blood vessels, inhibition of vascular inflammation and leukocyte adhesion, while nNOS regulates synaptic transmission in the central nervous system (CNS) and is present in smooth muscles promoting vasoregulation. The expression of iNOS occurs specially under inflammatory conditions through immunological or microbial stimuli (Förstermann and Sessa, 2012).

Post mortem analyses, clinical findings and experimental models of parkinsonism suggest the involvement of NO in the neurodegeneration observed in PD. Barthwal et al. (2001) demonstrated that the use of a non-specific inhibitor for NO (Nw-nitro-arginine-methyl-ester or L-NAME) results in a decrease in the neurodegenerative response after 6-OHDA injury in rats (Barthwal et al., 2001). In the MPTP model, inhibitors of nNOS—7-Nitroindazole (7-NI) and of iNOS—S-methylisothiourea (SMT) were effective in protecting against the neurotoxic effects of the lesion, suggesting the participation of NO in PD (Schulz et al., 1995; Di Monte et al., 1996; Aras et al., 2014). The administration of GW274150 [(2-((1-iminoethyl)amino)ethyl]-L-homocysteine ​​- selective iNOS inhibitor) after unilateral 6-OHDA injury significantly attenuated the loss of DA in the striatum of Sprague-Dawley rats (Broom et al., 2011). Furthermore, the increased degeneration of dopaminergic neurons in 6-OHDA-injured rats leads to a decrease in the concentration of nNOS in the substantia nigra of these animals (Czarnecka et al., 2013), while MPTP injury in mice results in an increase in iNOS (Liberatore et al., 1999). Literature showed differences in the effects of MPTP toxicity between males and females, with males having a faster decrease in DA and later, females having better recovery from this decrease. Furthermore, males were the first to show increased expression of iNOS and nNOS in the striatum (Joniec et al., 2009)

Another factor that suggests the involvement of NOS is the correlation between alterations in the genes responsible for iNOS and nNOS and the risk of developing PD (Levecque et al., 2003). Regarding peripheral NO, Çubukçu et al. (2016) demonstrate a decrease in plasma NO concentration, without a decrease in NOS activity in patients with PD. Thus, evidence indicates that NOS appear to participate in the neurodegeneration of dopaminergic neurons in animal models of PD. Therefore, the use of NOS inhibitors in the treatment of PD has been discussed (Broom et al., 2011).

The mechanisms by which NO contributes to neurodegeneration are not well understood. It is known that the induction of PD is related to increased oxidative stress in the brain, which leads to damage to the neurons of the substantia nigra (Dias et al., 2013). Studies suggest that the combination of NO with superoxides formed through the process of oxidative stress results in the formation of peroxynitrites (ONOO-), a molecule that is highly harmful to nervous tissue. In addition, NO has a high affinity for the heme group present in oxygen-carrying proteins, causing a delay in the functioning of the mitochondrial respiratory chain and consequently neuronal death (McDonald and Murad, 1996; Przedborski et al., 1996). Oxidative stress, in turn, decreases the availability of NO in endothelial cells and the central nervous system (Tieu et al., 2003). NO appears to be also involved in the dopamine metabolism by a common toxic pathway that involves mitochondrial compromise, nitroxidative stress and GSH depletion and that may be operating and contributing to the neurodegeneration observed in this disease (Nunes and Laranjinha, 2021).

## Nitric Oxide in Cardiovascular Dysfunction in Parkinson's Disease

Peripherally, NO acts as a potent vasodilator to increase blood flow, and it synthesis and bioavailability by the vascular endothelium contributes to greater vasodilation for adaptation in different conditions by the cardiovascular system (Green et al., 2004; McAllister and Laughlin, 2006; Di Francescomarino et al., 2009). Changes in its production can hamper hemodynamic responses and compromise cardiovascular health.

To date, few studies have evaluated NO in the cardiovascular system of animals with parkinsonism de Jager et al. (2018) observed differences in tissue concentrations of nitrite in sedentary animals or animals submitted to physical training before 6-OHDA injury, with NO being increased in the aorta of 6-OHDA animals compared to their controls. However, inhibition of NOS by L-NAME administration promoted a smaller increase in MAP in 6-OHDA animals, suggesting that eNOS nitrergic tonus is lower in animals injured by 6-OHDA than in Sham animals (de Jager et al., 2018). In this sense, it is possible that different isoforms are participating in these responses. Ongoing studies assess which isoform may be involved in vascular alterations in 6-OHDA animals and may contribute to explain the basal hypotension and sympathetic decrease in male rats.

Data from our group using female rats observed that adult females do not present the cardiovascular dysfunction observed by males, who have hypotension compared to their control group. However, when the females are submitted to ovariectomy they show basal hypotension, and a higher concentration of nitrite in the aorta, suggesting a role for NO associated with ovarian hormones at the vascular level (de Campos et al., 2020). Our group has studied the possible mechanisms of gender involvement in the cardiovascular dysfunctions of PD, including redox balance.

**FIGURE 1:**
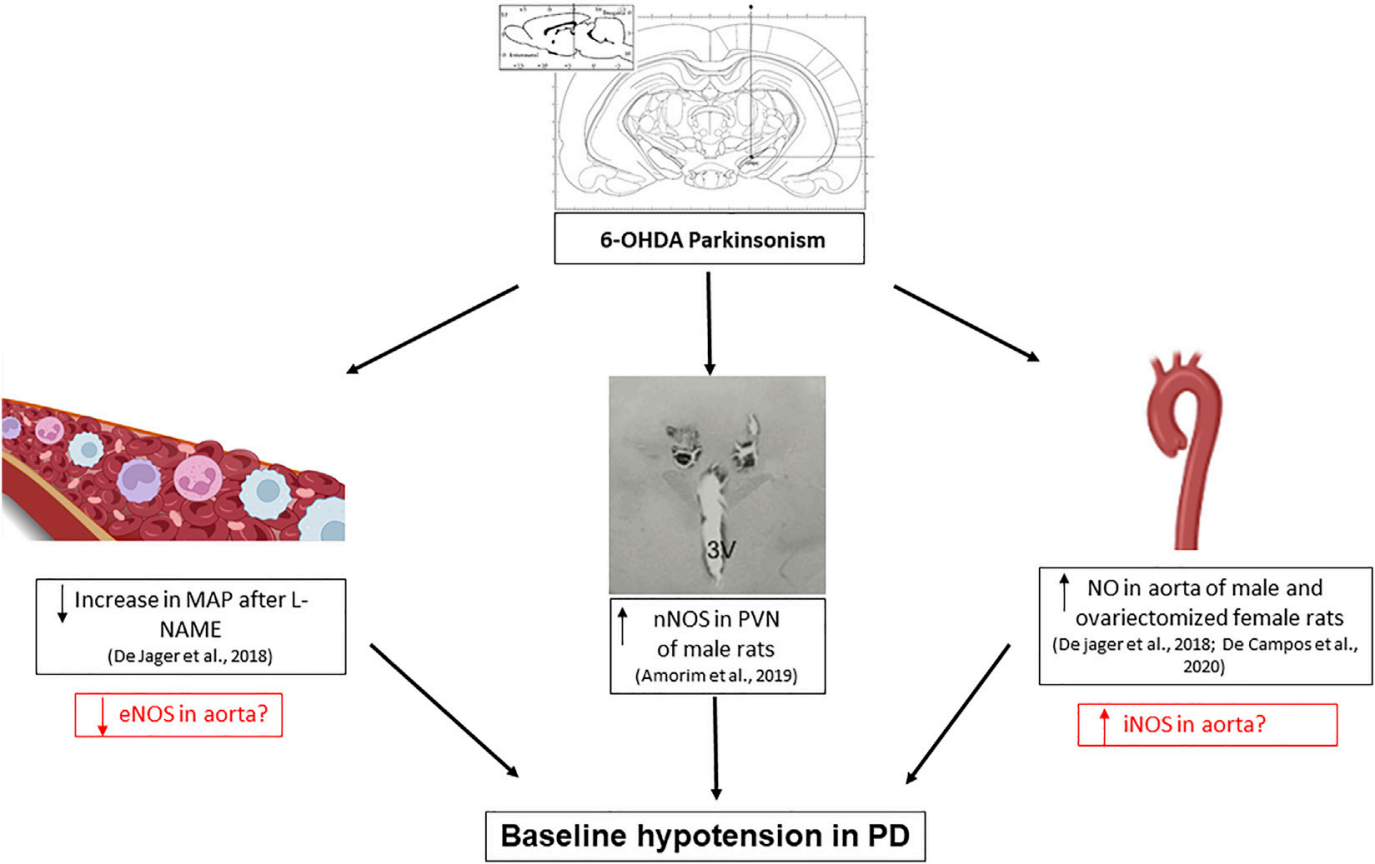
Scheme of changes involving nitric oxide alterations found in the cardiovascular system of rats induced to Parkinsonism using the bilateral model of substantia nigra pars compacta lesion with 6-hydroxydopamine (6-OHDA). The increased production of nitrite in the aorta of male and ovariectomized female rats, associated with an increase in nNOS activity in the region of the paraventricular nucleus of the hypothalamus (PVN), and a smaller increase in mean arterial pressure (MAP) after intravenous administration of L-NAME, suggests the involvement of the 3 isoforms of NO-synthase (NOS) in blood pressure dysfunction in PD. The boxes in red are questions and possible hypotheses. The central illustrations were taken from the cited articles by Ariza et al., 2015b and Turossi Amorim et al., 2019. The lateral illustrations were taken from BioRender.

Regarding the central control of the cardiovascular system, it was observed that nNOS is increased in the paraventricular nucleus of the hypothalamus, an area of cardiovascular control, leading to an increase in GABAergic tone on cardiovascular function and contributing to the lower basal BP values in male 6-OHDA injured rats (Turossi Amorim et al., 2019). By this way, the decreased baseline blood pressure in animals with Parkinsonism by 6-OHDA may be due to a central effect mediated by the NO in the PVN. However, we do not rule out the hypothesis of peripheral mechanisms participating in these cardiovascular dysfunctions (Figure 1). The study of these mechanisms is important to focus on future therapeutic targets for patients with PD.

## Conclusion

The findings so far regarding the participation of NO in the cardiovascular dysfunctions in PD show that hypothalamic nuclei that participate in tonic autonomic and cardiovascular control seem to be a target for NO alterations. In addition, still initial data have suggested the participation of NO peripherally (blood vessels), which brings us perspectives about possible new treatments for the cardiovascular dysfunctions in PD, especially related to hypotension.
